# Visfatin increases ICAM-1 expression and monocyte adhesion in human osteoarthritis synovial fibroblasts by reducing miR-320a expression

**DOI:** 10.18632/aging.103889

**Published:** 2020-09-29

**Authors:** Yat-Yin Law, Yu-Min Lin, Shan-Chi Liu, Min-Huan Wu, Wen-Hui Chung, Chun-Hao Tsai, Yi-Chin Fong, Chih-Hsin Tang, Chin-Kun Wang

**Affiliations:** 1 Institute of Medicine, Chung Shan Medical University, Taichung, Taiwan; 2 Department of Orthopedics, Chung Shan Medical University Hospital, Taichung, Taiwan; 3 Department of Orthopedic Surgery, Taichung Veterans General Hospital, Taichung, Taiwan; 4 Department of Medical Education and Research, China Medical University Beigang Hospital, Yunlin, Taiwan; 5 Physical Education Office, Tunghai University, Taichung, Taiwan; 6 Sports Recreation and Health Management Continuing Studies, Tunghai University, Taichung, Taiwan; 7 Department of Pharmacology, School of Medicine, China Medical University, Taichung, Taiwan; 8 Department of Sports Medicine, College of Health Care, China Medical University, Taichung, Taiwan; 9 Department of Orthopedic Surgery, China Medical University Hospital, Taichung, Taiwan; 10 Department of Orthopedic Surgery, China Medical University Beigang Hospital, Yunlin, Taiwan; 11 Chinese Medicine Research Center, China Medical University, Taichung, Taiwan; 12 Department of Biotechnology, College of Health Science, Asia University, Taichung, Taiwan; 13 School of Nutrition, Chung Shan Medical University, Taichung, Taiwan

**Keywords:** osteoarthritis, visfatin, ICAM-1, miR-320a, monocytes

## Abstract

Pathophysiological events that modulate the progression of structural changes in osteoarthritis (OA) include monocyte adhesion and infiltration, and synovial inflammation. In particular, the adhesion protein intercellular adhesion molecule type 1 (ICAM-1) promotes monocyte recruitment into the synovial tissue. Visfatin is an adipocyte hormone that promotes the release of inflammatory cytokines during OA progression. We report that visfatin enhances ICAM-1 expression in human OA synovial fibroblasts (OASFs) and facilitates the adhesion of monocytes with OASFs. AMPK and p38 inhibitors, as well as their respective siRNAs, attenuated the effects of visfatin upon ICAM-1 synthesis and monocyte adhesion. We also describe how miR-320a negatively regulates visfatin-induced promotion of ICAM-1 expression and monocyte adhesion. We detail how visfatin affects ICAM-1 expression and monocyte adhesion with OASFs by inhibiting miR-320a synthesis via the AMPK and p38 signaling pathways.

## INTRODUCTION

Synovial inflammation is an important contributor to the pathogenesis of osteoarthritis (OA) [1], with the synthesis of chondrolytic enzymes and proinflammatory mediators by the inflamed synovium eroding cartilage and enhancing the inflammatory process [2, 3]. Halting the excretion of chondrolytic enzymes and inflammatory mediators by OA synovial fibroblasts (OASFs) is expected to mitigate OA disease [2, 4–7].

Infiltration of mononuclear cells to the inflammatory sites and their adhesion to the synovium membrane are critical steps during OA progression [8]. Several different adhesion molecules regulate monocyte migration and adhesion in the articular microenvironment during OA development [8, 9], including intercellular adhesion molecule-1 (ICAM-1) [10]. ICAM-1 is a critical modulator of monocyte recruitment into the synovial tissue and high levels of ICAM-1 expression have been found in the synovium of OA patients [11, 12]. Downregulation of ICAM-1 expression in synovial fluid is suggested to be an effective method for inhibiting inflammatory activity and ameliorating symptoms in OA [13, 14].

Micro RNAs (miRNAs) control the synthesis of target genes at the post-transcriptional level and inhibit the expression of target genes [15]. Numerous miRNAs regulate OA pathogenesis [16], although it is not clear as to how miRNAs regulate ICAM-1 expression in OA. The proinflammatory adipokine visfatin is produced by visceral white adipose tissue in the bone marrow, skeletal muscles and liver [17]. By stimulating the expression of interleukin 6 (IL-6) and tumor necrosis factor alpha (TNF-α) in OASFs, visfatin contributes to synovial joint damage [18]. It is not known how visfatin affects ICAM-1-dependent monocyte adhesion during OA progression. We describe how visfatin increases monocyte adhesion to OASFs by increasing ICAM-1 expression. The reduction in miR-320a expression via the adenosine monophosphate-activated protein kinase (AMPK) and p38 signaling pathways is mediated by the effects of visfatin, indicating that this adipokine may be an appropriate therapeutic target in OA.

## RESULTS

### Visfatin and ICAM-1 expression are positively correlated in OA

**Figure 1:**
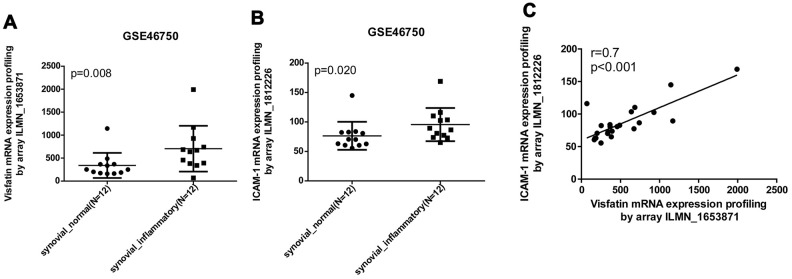
**Visfatin expression is positively correlated with ICAM-1 expression in inflammatory synovial tissues.** (**A**, **B**) Expression levels of visfatin and ICAM-1 in 12 paired normal and inflammatory synovial tissues retrieved from the GEO dataset (accession code: GSE46750). (**C**) Correlation between levels of visfatin and ICAM-1 expression in inflammatory synovial tissues. Mann-Whitney testing was applied in (**A**, **B**).

**Figure 2:**
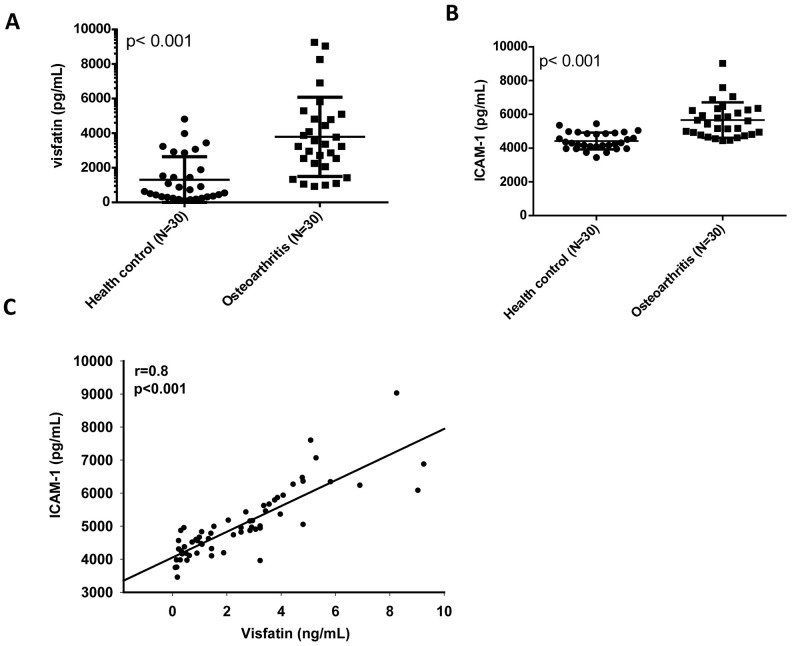
**Visfatin expression is positively correlated with ICAM-1 expression in OA patients.** (**A**, **B**) ELISA analysis showing higher serum visfatin and ICAM-1 levels among OA patients (n=30) compared with healthy controls (n=30). (**C**) Correlation between levels of visfatin and ICAM-1 expression in serum samples retrieved from OA patients. All ELISA procedures were independently repeated three times. Mann-Whitney testing was applied in (**A**, **B**).

Our analysis of records from the Gene Expression Omnibus (GEO) database revealed higher levels of visfatin and ICAM-1 expression in inflamed synovial tissue compared with normal synovial tissue (Figure 1A, 1B). A positive correlation was observed for levels of visfatin and ICAM-1 expression (Figure 1C). An ELISA assay confirmed significantly higher serum visfatin and ICAM-1 concentrations in patients with OA compared with healthy controls (Figure 2A, 2B). Serum visfatin and ICAM-1 concentrations were positively correlated (Figure 2C).

### Visfatin increases ICAM-1 expression and monocyte adhesion in OASFs

**Figure 3:**
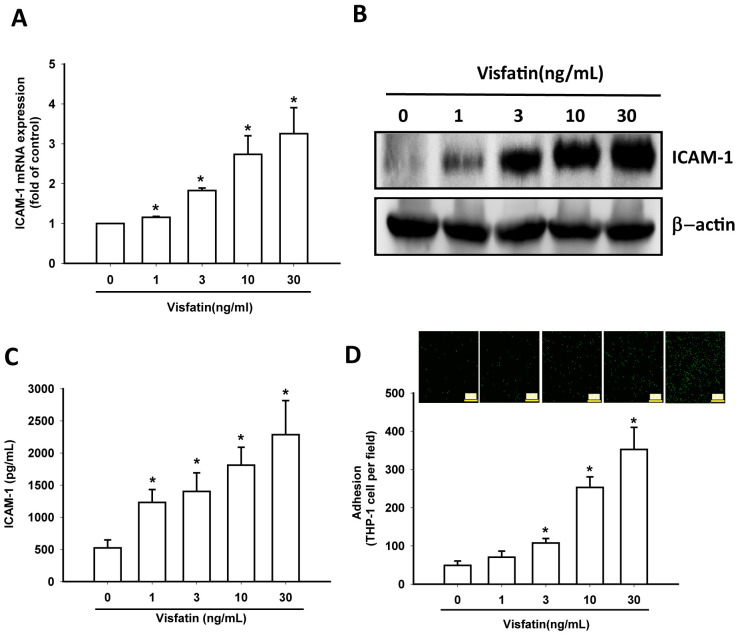
**Visfatin stimulates ICAM-1 expression and monocyte adhesion in OASFs.** (**A**–**C**) OASFs were incubated with visfatin (1–30 ng/mL) for 24 h, and ICAM-1 expression was examined by RT-qPCR, Western blot and ELISA analysis. (**D**) OASFs were incubated with visfatin (1–30 ng/mL) for 24 h. THP-1 cells loaded with BCECF-AM were added to OASFs for 6 h, then THP-1 cell adherence was measured by fluorescence microscopy. * *p*<0.05 compared with the control group.

In this study, visfatin (1–30 ng/mL) dose-dependently stimulated transcription of ICAM-1 mRNA and also increased the translation of ICAM-1 at the protein level (Figure 3A, 3B) and also the excretion of ICAM-1 protein by OASFs (Figure 3C). Visfatin markedly increased the adhesiveness between OASFs and monocytes (THP-1 cells) in a concentration-dependent manner (Figure 3D), indicating that visfatin promotes ICAM-1 expression and monocyte adhesion in human OASFs.

### Visfatin promotes ICAM-1 expression and monocyte adhesion via the AMPK and p38 signaling pathways

**Figure 4:**
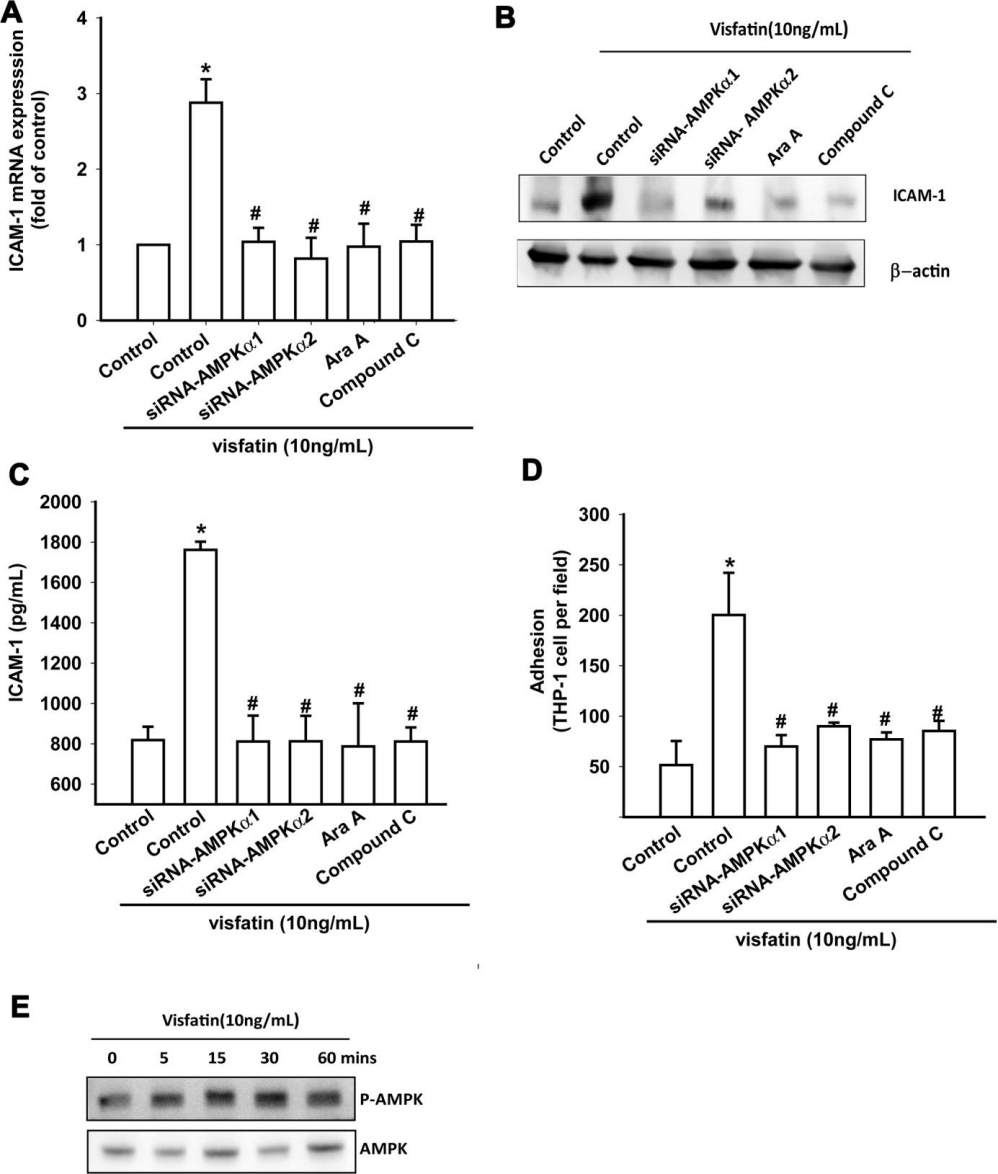
**The AMPK pathway is involved in visfatin-induced ICAM-1 synthesis and monocyte adhesion.** (**A**–**C**) OASFs were pretreated with AMPK inhibitors (Ara A, compound C) or transfected with AMPK siRNAs, then incubated with visfatin for 24 h. ICAM-1 levels were examined by RT-qPCR, Western blot and ELISA assays. (**D**) OASFs were treated with the same conditions as those described in (**A**). THP-1 cells loaded with BCECF-AM were added to OASFs for 6 h, then THP-1 cell adherence was measured by fluorescence microscopy. (**E**) OASFs were incubated with visfatin for the indicated time intervals, and AMPK phosphorylation was examined by Western blot. * *p*<0.05 compared with the control group; ^#^ *p*<0.05 compared with the visfatin-treated group.

OASFs were pretreated with AMPK inhibitors (Ara A and compound C) or were transfected with AMPKα1 and AMPKα2 small interfering RNAs (siRNAs). RT-qPCR, Western blot and ELISA assays confirmed that the AMPK inhibitors and AMPK siRNAs significantly reduced visfatin-increased ICAM-1 synthesis in OASFs (Figure 4A–4C). These compounds also mitigated monocyte adhesion to OASFs (Figure 4D). In Western blot analysis, visfatin time-dependently promoted AMPK phosphorylation (Figure 4E).

**Figure 5:**
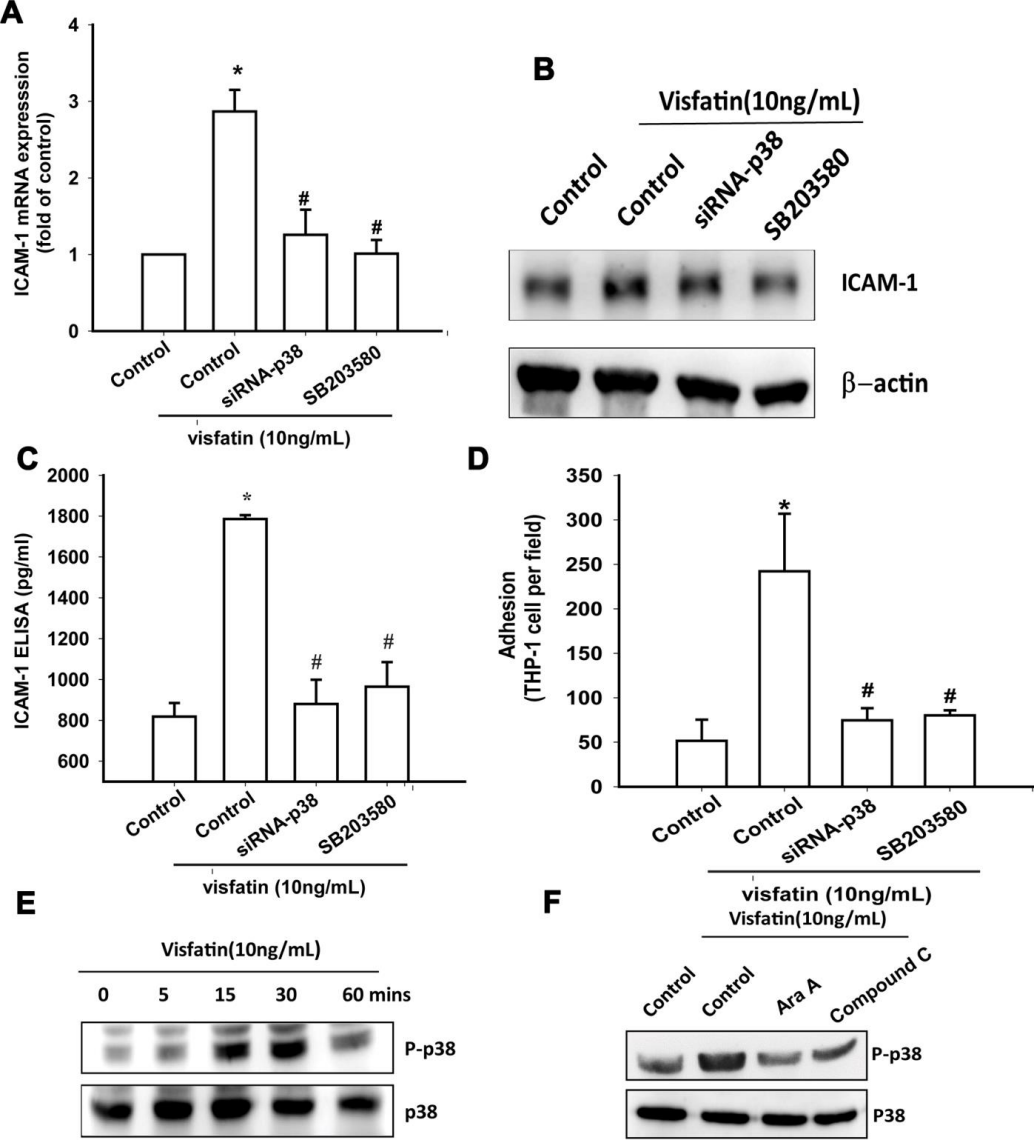
**The p38 pathway is involved in visfatin-induced ICAM-1 synthesis and monocyte adhesion.** (**A**–**C**) OASFs were pretreated with a p38 inhibitor (SB203580) or transfected with p38 siRNA, then incubated with visfatin for 24 h. ICAM-1 levels were examined by RT-qPCR, Western blot and ELISA assays. (**D**) OASFs were treated with the same conditions as those described in (**A**). THP-1 cells loaded with BCECF-AM were added to OASFs for 6 h, then THP-1 cell adherence was measured by fluorescence microscopy. (**E, F**) OASFs were incubated with visfatin for the indicated time intervals or pretreated with AMPK inhibitors then stimulated with visfatin, and p38 phosphorylation was examined by Western blot. * *p*<0.05 compared with the control group; ^#^ *p*<0.05 compared with the visfatin-treated group.

Treatment of OASFs with a p38 inhibitor (SB203580) or transfection of OASFs with p38 siRNA prior to visfatin stimulation markedly diminished visfatin-induced increases in ICAM-1 expression and monocyte adhesion (Figure 5A–5D). In Western blot analysis, the time-dependent promotion of p38 phosphorylation by visfatin (Figure 5E) was reduced by AMPK inhibitors (Figure 5F). These findings suggest that visfatin facilitates ICAM-1 production and monocyte adhesion in human OASFs through the AMPK and p38 signaling pathways.

### Visfatin increases ICAM-1 production and monocyte adhesion via the inhibition of miR-320a synthesis

**Figure 6:**
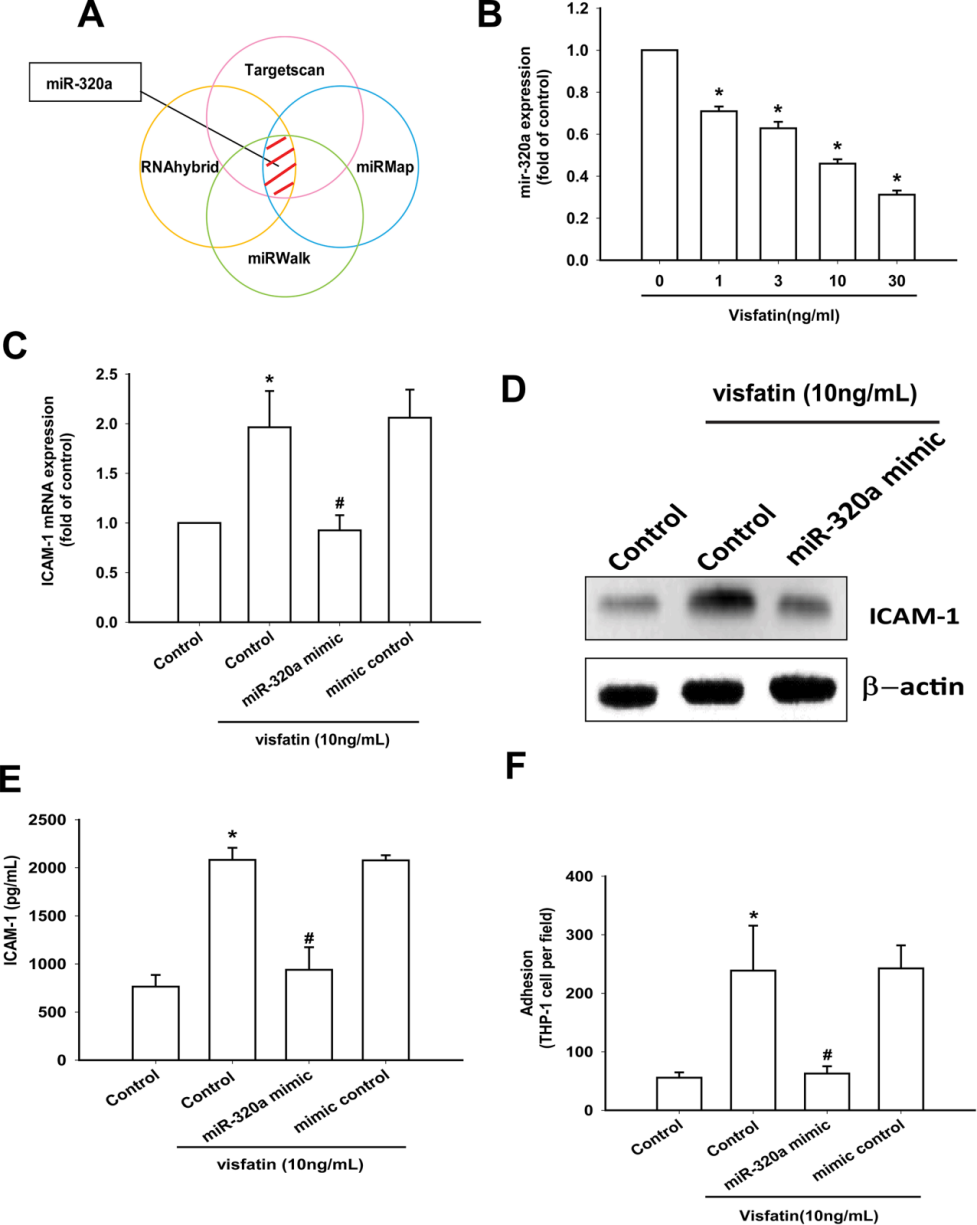
**Visfatin promotes ICAM-1 production and monocyte adhesion by suppressing miR-320a.** (**A**) Open-source software (TargetScan, miRMap, RNAhybrid, and miRWalk) was used to identify which miRNAs could possibly interfere with ICAM-1 transcription. (**B**) OASFs were incubated with visfatin (1–30 ng/mL). miR-320a expression was examined by RT-qPCR. (**C**–**E**) OASFs were transfected with miR-144-3p mimic and mimic control (serving as the vehicle control) and then stimulated with visfatin. ICAM-1 levels were examined by RT-qPCR, Western blot and ELISA assays. (**F**) OASFs were treated with the same conditions as those described in (**C**). THP-1 cells loaded with BCECF-AM were added to OASFs for 6 h, then THP-1 cell adherence was measured by fluorescence microscopy. * *p*<0.05 compared with the control group; ^#^ *p*<0.05 compared with the visfatin-treated group.

Open-source software (TargetScan, miRMap, RNAhybrid, and miRWalk) suggested that miR-320a interferes with ICAM-1 transcription (Figure 6A). Visfatin treatment of OASFs concentration-dependently reduced miR-320a expression (Figure 6B). ICAM-1 expression and monocyte adhesion in visfatin-treated OASFs was lower after transfection with miR-320a mimic compared with after mimic control (serving as the vehicle control) (Figure 6C–6F).

**Figure 7:**
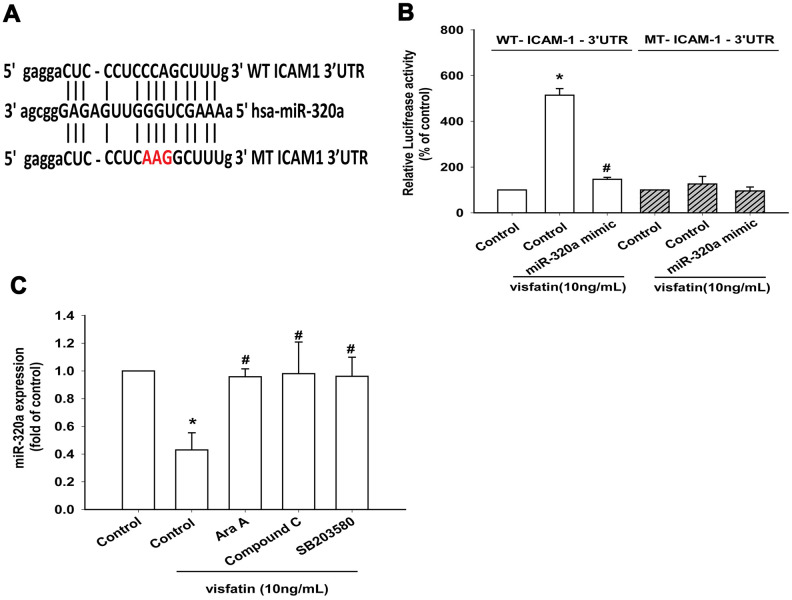
**Visfatin suppresses miR-320a synthesis via the AMPK and p38 pathways.** (**A**) Schematic 3′-UTR representation of human ICAM-1 containing the miR-320a binding site. (**B**) OASFs were transfected with the indicated luciferase plasmid with or without miR-320a mimic, then stimulated with visfatin. Relative luciferase activity was examined. (**C**) OASFs were pretreated with Ara A, compound C and SB203580 for 30 min, then incubated with visfatin for 24 h. The expression of miR-320a was examined by qPCR. * *p*<0.05 compared with the control group; ^#^ *p*<0.05 compared with the visfatin-treated group.

**Figure 8:**
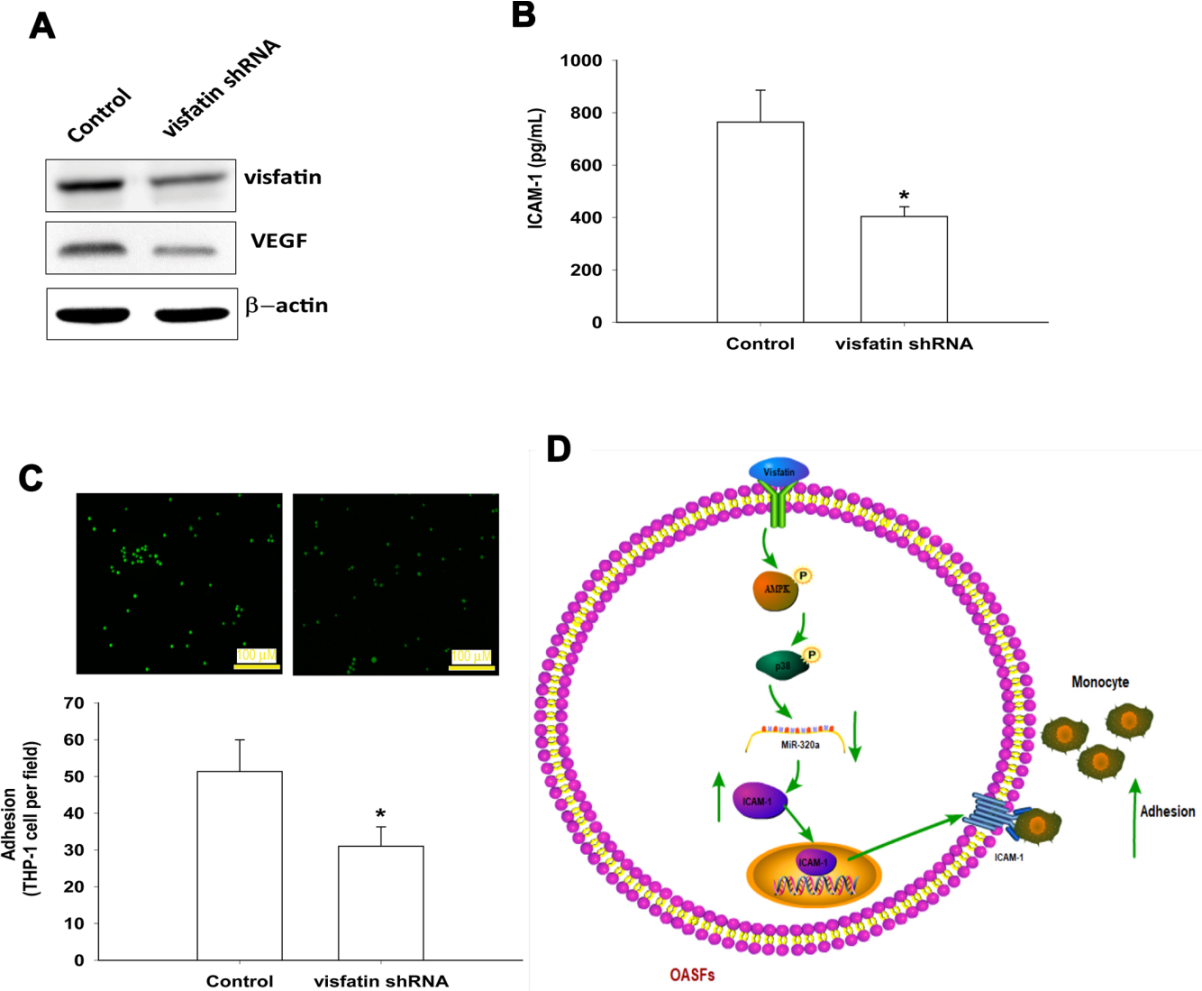
**Knockdown visfatin reduces ICAM-1 expression and monocyte adhesion in OASFs.** (**A**, **B**) OASFs were transfected with visfatin shRNA. Visfatin and ICAM-1 expression was examined by Western blot and ELISA. (**C**) OASFs were treated with the same conditions as those described in (**A**). THP-1 cells loaded with BCECF-AM were added to OASFs for 6 h, then THP-1 cell adherence was measured by fluorescence microscopy. (**D**) The schematic diagram summarizes the mechanism whereby visfatin promotes ICAM-1 expression and monocyte adhesion in OASFs. Visfatin promotes ICAM-1 expression and enhances monocyte adhesion to OASFs by downregulating miR-320a through the AMPK and p38 signaling pathways. * *p*<0.05 compared with the control group.

The luciferase reporter vector with the wild-type 3’UTR of ICAM-1 mRNA (wt-ICAM-1-3’UTR) and a mutated vector harboring mismatches in the predicted miR-320a binding site (mut-ICAM-1-3’UTR) were used to determine whether miR-320a controls transcription of the *ICAM-1* gene (Figure 7A). The miR-320a mimic inhibited visfatin-increased luciferase activity in the wt- ICAM-1-3’UTR plasmid only (Figure 7B). AMPK and p38 inhibitors reversed the effects of visfatin on miR-320a expression (Figure 7C). These results suggest that visfatin inhibits miR-320a expression via the AMPK and p38 signaling pathways. Visfatin shRNA reduced visfatin and ICAM-1 expression in OASFs (Figure 8A, 8B) and also monocyte adhesion (Figure 8C), confirming that visfatin regulates the expression of ICAM-1 and adhesion of monocytes in OASFs.

## DISCUSSION

OA pathogenesis remains poorly understood, although it is established that synovium inflammation has a pivotal part in its pathogenesis [19], which highlights the importance of synovium-targeted therapy in this disease [6, 20]. Adhesion molecules in the synovial lining assist with monocyte infiltration into inflamed OA synovium [14]. In this study, we found that ICAM-1 acts as a target protein for visfatin and facilitates monocyte adhesion to OASFs. We also found that visfatin enhances ICAM-1 production by inhibiting miR-320a expression via the AMPK and p38 signaling pathways, and facilitates monocyte adhesion to human OASFs.

Previous findings of higher visfatin concentrations in synovial fluid from OA patients compared with healthy synovial fluid [21, 22] were confirmed in this study. Our analysis of records from the GEO database also found higher visfatin levels in inflammatory synovial tissue than in normal synovial tissue. Those records and our study findings revealed positive correlations between visfatin and ICAM-1 concentrations, highlighting the importance of visfatin as a molecular target in OA therapy. Adiponectin and leptin are also key adipokines in OA disease [23]. Adiponectin has been documented to promote inflammatory cytokine release and matrix metalloproteinase production in OASFs [24, 25], while leptin increases inflammatory cytokine production and ADAM expression during OA pathogenesis [26–28]. Clearly, visfatin, adiponectin and leptin play critical roles in OA disease. We have also confirmed novel functioning of visfatin that is similar to activities of the previously identified adipokines in OA pathogenesis.

Activation of AMPK signaling regulates multiple cellular functions [29], including the expression of adhesion molecules [30, 31]. We have found that visfatin facilitates AMPK phosphorylation, while AMPK inhibitors and siRNAs attenuate visfatin-enhanced ICAM-1 production and monocyte adhesion to OASFs. AMPK-dependent p38 activation is critical for controlling cell adhesion and motility [30, 32]. Some papers have mentioned that p38 activates AMPK and is upstream of AMPK [31]. Thus, our evidence indicates that visfatin facilitates p38 phosphorylation, and that this is reversed by AMPK inhibitors. Our data therefore confirms that AMPK is upstream of p38 and that p38 is required for visfatin-promoted ICAM-1 production and monocyte adhesion.

Regulating miRNA expression should help to lessen OA inflammation [33, 34]. Open-source miRNA software identified that miR-320a potentially interferes with ICAM-1 transcription, which was supported by our findings showing that visfatin reduced miR-320a synthesis, while overexpression of miR-320a mimic mitigated the stimulatory effects of visfatin on ICAM-1 expression and monocyte adhesion. It appears that visfatin facilitates ICAM-1 production and monocyte adhesion by reducing miR-320a expression through AMPK and p38 signaling.

A limitation of our research is that demographic details and any other general information of our study participants were not recorded, to maintain patient confidentiality. Thus, we could not compare demographic details with levels of visfatin and ICAM-1 expression.

Previous research has described how 3 months of garlic supplementation in postmenopausal overweight or obese women with knee OA was associated with significant reductions from baseline in resistin levels and also pain scores, which suggests that effectively lowering concentrations of a proinflammatory adipocytokine such as resistin may reduce pain severity [35]. Our study focused on whether changes in visfatin concentrations induce changes in monocyte adhesion to the synovium and consequently the severity of OA disease. We observed that knockdown of visfatin resulted in lower levels of monocyte adhesion, which may attenuate OA disease. For the purposes of this study, we did not seek to determine levels of any other proinflammatory cytokines, such as TNF-α. More research is needed in this area.

In summary, visfatin increased ICAM-1 expression and promoted monocyte adhesion to OASFs by inhibiting miR-320a synthesis through the AMPK and p38 signaling pathways (Figure 8D). Targeting visfatin may improve the pathogenesis of OA.

## MATERIALS AND METHODS

Antibodies against ICAM-1 (SC-107), p-AMPK (SC-33524), AMPK (SC-25792), p-p38 (SC-166182), p38 (SC-271120) and β-actin (SC-47778) were all bought from Santa Cruz (Santa Cruz, CA, USA). All ON-TARGET*plus* siRNAs were purchased from Dharmacon (Lafayette, CO, USA). Cell culture supplements were purchased from Invitrogen (Carlsbad, CA, USA). A Dual-Luciferase^®^ Reporter Assay System was bought from Promega (Madison, WI, USA). qPCR primers and probes, as well as the Taqman^®^ one-step PCR Master Mix, were supplied by Applied Biosystems (Foster City, CA, USA). All other chemicals not mentioned above were supplied by Sigma-Aldrich (St. Louis, MO, USA).

### Cell culture

Synovial tissue from the suprapatellar pouch of the knee was obtained from patients whose radiographically-detected OA of the knee was classified under the Ahlbäck criteria as stage IV OA [36]. Synovial fibroblasts were cultured in DMEM medium supplemented with 10% fetal bovine serum (FBS), 50 units/mL penicillin and 50 μg/mL streptomycin, as previously described [37, 38].

THP-1, a human leukemia cell line of monocyte/macrophage lineage, was obtained from the American Type Culture Collection (Manassas, VA, USA) and grown in RPMI-1640 medium containing 10% FBS.

### Clinical samples

Clinical samples were collected from patients meeting the following inclusion criteria: (1) aged over 20 years presenting with an accidental or sports injury requiring joint replacement and repair; or (2) degenerative arthritis. Exclusion criteria specified patients who did not satisfy either of these two categories. Serum (2 mL) and synovial tissue samples (which could be of unlimited sizes) were obtained from patients with OA undergoing knee replacement surgery and also from those undergoing arthroscopy after trauma/joint derangement (who served as healthy controls) at China Medical University Hospital, Taichung, Taiwan. The study protocol was approved by the Institutional Review Board (IRB) of China Medical University Hospital and all methods were performed in accordance with the IRB’s guidelines and regulations. Informed written consent was obtained from all patients.

### Real-time quantitative PCR analysis of mRNA and miRNA

Total RNA was extracted from OASFs by TRIzol; reverse transcription used 1 μg of total RNA transcribed into cDNA by oligo (dT) primers. RT-qPCR used the Taqman^®^ One-Step RT-PCR Master Mix. All RT-qPCR assays were performed using the StepOnePlus sequence detection system (Applied Biosystems) [39, 40].

### Western blot analysis

Cell lysate was separated by SDS-PAGE electrophoresis then transferred to polyvinylidene difluoride (PVDF) membranes, following the method described in our previous work [41, 42]. After blocking the blots with 4% bovine serum albumin, the blots were treated with primary antibody and then secondary antibody. Enhanced chemiluminescent imaging of the blots was visualized with the UVP Biospectrum system (UVP, Upland, CA, USA) [43–45].

### ELISA assay

OASFs were cultured in 24-well plates until they reached 90% confluence, when they were then changed to serum-free medium, in which they were treated with visfatin for 24 h with or without the transfection of siRNAs or inhibitors. The CM was collected and ICAM-1 levels were quantified with the ICAM-1 ELISA kit.

Serum was collected from patients with OA or normal healthy controls. Visfatin and ICAM-1 levels were quantified by the Visfatin ELISA kit (EIA-VIS-1; RayBiotech, Peachtree corners, GA, USA; detection ranges 100–1,000,000 pg/mL) and the ICAM-1 ELISA kit (DY720; R&D Systems, Minneapolis, MN, USA).

### Analysis of the GEO database

Data on visfatin and ICAM-1 mRNA expression for normal healthy controls and OA patients were retrieved from the GEO dataset records [46].

### Luciferase assays

Wild-type and mutant ICAM-1 3’-UTR plasmids were purchased from Invitrogen (Carlsbad, CA, USA). Luciferase activity was assayed using the method described in our previous publications [2, 37, 47].

### Cell adhesion assay

THP-1 cells were loaded with BCECF-AM (10 μM) for 1 h at 37°C in RPMI-1640 medium and subsequently washed by centrifugation. OASFs grown on glass coverslips were incubated with visfatin then incubated with THP-1 cells at 37°C for 1 h. Nonadherent THP-1 cells were removed and gently washed with PBS. The number of adherent THP-1 cells was counted using a fluorescent microscope.

### Statistics

All values are given as the mean ± standard error of the mean ± S.D. All ELISA procedures were repeated three times. The Student’s *t*-test assessed between-group differences. A *p* value of <0.05 was considered to be statistically significant.

## References

[1] Yuan XL, Meng HY, Wang YC, Peng J, Guo QY, Wang AY, Lu SB. Bone-cartilage interface crosstalk in osteoarthritis: potential pathways and future therapeutic strategies. Osteoarthritis Cartilage. 2014; 22:1077–89. 10.1016/j.joca.2014.05.023 24928319

[2] Kuo SJ, Yang WH, Liu SC, Tsai CH, Hsu HC, Tang CH. Transforming growth factor β1 enhances heme oxygenase 1 expression in human synovial fibroblasts by inhibiting microRNA 519b synthesis. PLoS One. 2017; 12:e0176052. 10.1371/journal.pone.0176052 28423042 PMC5397058

[3] Tang CH. Research of pathogenesis and novel therapeutics in arthritis. Int J Mol Sci. 2019; 20:1646. 10.3390/ijms20071646 30987068 PMC6479975

[4] Benito MJ, Veale DJ, FitzGerald O, van den Berg WB, Bresnihan B. Synovial tissue inflammation in early and late osteoarthritis. Ann Rheum Dis. 2005; 64:1263–67. 10.1136/ard.2004.025270 15731292 PMC1755629

[5] Dehghan M, Asgharian S, Khalesi E, Ahmadi A, Lorigooini Z. Comparative study of the effect of thymus daenensis gel 5% and diclofenac in patients with knee osteoarthritis. Biomedicine (Taipei). 2019; 9:9. 10.1051/bmdcn/2019090209 31124455 PMC6533939

[6] Sellam J, Berenbaum F. The role of synovitis in pathophysiology and clinical symptoms of osteoarthritis. Nat Rev Rheumatol. 2010; 6:625–35. 10.1038/nrrheum.2010.159 20924410

[7] Xu ZY, Liu YL, Lin JB, Cheng KL, Wang YG, Yao HL, Wei-Peng, Wu HY, Su WW, Shaw PC, Li PB. Preparative expression and purification of a nacreous protein N16 and testing its effect on osteoporosis rat model. Int J Biol Macromol. 2018; 111:440–45. 10.1016/j.ijbiomac.2018.01.054 29329805

[8] Zhang H, Cai D, Bai X. Macrophages regulate the progression of osteoarthritis. Osteoarthritis Cartilage. 2020; 28:555–61. 10.1016/j.joca.2020.01.007 31982565

[9] Smolen JS, Aletaha D, McInnes IB. Rheumatoid arthritis. Lancet. 2016; 388:2023–38. 10.1016/S0140-6736(16)30173-8 27156434

[10] Ramos TN, Bullard DC, Barnum SR. ICAM-1: isoforms and phenotypes. J Immunol. 2014; 192:4469–74. 10.4049/jimmunol.1400135 24795464 PMC4015451

[11] Köller M, Aringer M, Kiener H, Erlacher L, Machold K, Eberl G, Studnicka-Benke A, Graninger W, Smolen J. Expression of adhesion molecules on synovial fluid and peripheral blood monocytes in patients with inflammatory joint disease and osteoarthritis. Ann Rheum Dis. 1999; 58:709–12. 10.1136/ard.58.11.709 10531076 PMC1752805

[12] Lavigne P, Benderdour M, Lajeunesse D, Shi Q, Fernandes JC. Expression of ICAM-1 by osteoblasts in healthy individuals and in patients suffering from osteoarthritis and osteoporosis. Bone. 2004; 35:463–70. 10.1016/j.bone.2003.12.030 15268898

[13] Karatay S, Kiziltunc A, Yildirim K, Karanfil RC, Senel K. Effects of different hyaluronic acid products on synovial fluid levels of intercellular adhesion molecule-1 and vascular cell adhesion molecule-1 in knee osteoarthritis. Ann Clin Lab Sci. 2004; 34:330–35. 15487709

[14] Lavigne P, Benderdour M, Shi Q, Lajeunesse D, Fernandes JC. Involvement of ICAM-1 in bone metabolism: a potential target in the treatment of bone diseases? Expert Opin Biol Ther. 2005; 5:313–20. 10.1517/14712598.5.3.313 15833069

[15] Lee RC, Feinbaum RL, Ambros V. The C. Elegans heterochronic gene lin-4 encodes small RNAs with antisense complementarity to lin-14. Cell. 1993; 75:843–54. 10.1016/0092-8674(93)90529-y 8252621

[16] Asahara H. Current status and strategy of microRNA research for cartilage development and osteoarthritis pathogenesis. J Bone Metab. 2016; 23:121–27. 10.11005/jbm.2016.23.3.121 27622175 PMC5018604

[17] Tilg H, Moschen AR. Adipocytokines: mediators linking adipose tissue, inflammation and immunity. Nat Rev Immunol. 2006; 6:772–83. 10.1038/nri1937 16998510

[18] Wu MH, Tsai CH, Huang YL, Fong YC, Tang CH. Visfatin promotes IL-6 and TNF-α production in human synovial fibroblasts by repressing miR-199a-5p through ERK, p38 and JNK signaling pathways. Int J Mol Sci. 2018; 19:190. 10.3390/ijms19010190 29316707 PMC5796139

[19] Gao B, Gao W, Wu Z, Zhou T, Qiu X, Wang X, Lian C, Peng Y, Liang A, Qiu J, Zhu Y, Xu C, Li Y, et al. Melatonin rescued interleukin 1β-impaired chondrogenesis of human mesenchymal stem cells. Stem Cell Res Ther. 2018; 9:162. 10.1186/s13287-018-0892-3 29898779 PMC6001057

[20] Mathiessen A, Conaghan PG. Synovitis in osteoarthritis: current understanding with therapeutic implications. Arthritis Res Ther. 2017; 19:18. 10.1186/s13075-017-1229-9 28148295 PMC5289060

[21] Liao L, Chen Y, Wang W. The current progress in understanding the molecular functions and mechanisms of visfatin in osteoarthritis. J Bone Miner Metab. 2016; 34:485–90. 10.1007/s00774-016-0743-1 26969394

[22] Fioravanti A, Giannitti C, Cheleschi S, Simpatico A, Pascarelli NA, Galeazzi M. Circulating levels of adiponectin, resistin, and visfatin after mud-bath therapy in patients with bilateral knee osteoarthritis. Int J Biometeorol. 2015; 59:1691–700. 10.1007/s00484-015-0977-y 25750093

[23] MᵃᶜDonald IJ, Liu SC, Huang CC, Kuo SJ, Tsai CH, Tang CH. Associations between adipokines in arthritic disease and implications for obesity. Int J Mol Sci. 2019; 20:1505. 10.3390/ijms20061505 30917508 PMC6471239

[24] Tang CH, Chiu YC, Tan TW, Yang RS, Fu WM. Adiponectin enhances IL-6 production in human synovial fibroblast via an AdipoR1 receptor, AMPK, p38, and NF-κB pathway. J Immunol. 2007; 179:5483–92. 10.4049/jimmunol.179.8.5483 17911635

[25] Koskinen A, Juslin S, Nieminen R, Moilanen T, Vuolteenaho K, Moilanen E. Adiponectin associates with markers of cartilage degradation in osteoarthritis and induces production of proinflammatory and catabolic factors through mitogen-activated protein kinase pathways. Arthritis Res Ther. 2011; 13:R184. 10.1186/ar3512 22077999 PMC3334633

[26] Tong KM, Shieh DC, Chen CP, Tzeng CY, Wang SP, Huang KC, Chiu YC, Fong YC, Tang CH. Leptin induces IL-8 expression via leptin receptor, IRS-1, PI3K, Akt cascade and promotion of NF-κB/p300 binding in human synovial fibroblasts. Cell Signal. 2008; 20:1478–88. 10.1016/j.cellsig.2008.04.003 18501560

[27] Yang WH, Liu SC, Tsai CH, Fong YC, Wang SJ, Chang YS, Tang CH. Leptin induces IL-6 expression through OBRl receptor signaling pathway in human synovial fibroblasts. PLoS One. 2013; 8:e75551. 10.1371/journal.pone.0075551 24086566 PMC3785513

[28] Yaykasli KO, Hatipoglu OF, Yaykasli E, Yildirim K, Kaya E, Ozsahin M, Uslu M, Gunduz E. Leptin induces ADAMTS-4, ADAMTS-5, and ADAMTS-9 genes expression by mitogen-activated protein kinases and NF-ĸB signaling pathways in human chondrocytes. Cell Biol Int. 2015; 39:104–12. 10.1002/cbin.10336 25045124

[29] Carling D. AMPK signalling in health and disease. Curr Opin Cell Biol. 2017; 45:31–37. 10.1016/j.ceb.2017.01.005 28232179

[30] Chen HT, Tsou HK, Chen JC, Shih JM, Chen YJ, Tang CH. Adiponectin enhances intercellular adhesion molecule-1 expression and promotes monocyte adhesion in human synovial fibroblasts. PLoS One. 2014; 9:e92741. 10.1371/journal.pone.0092741 24667577 PMC3965461

[31] Tang CH, Lu ME. Adiponectin increases motility of human prostate cancer cells via adipoR, p38, AMPK, and NF-κB pathways. Prostate. 2009; 69:1781–89. 10.1002/pros.21029 19676095

[32] Hsu CJ, Wu MH, Chen CY, Tsai CH, Hsu HC, Tang CH. AMP-activated protein kinase activation mediates CCL3-induced cell migration and matrix metalloproteinase-2 expression in human chondrosarcoma. Cell Commun Signal. 2013; 11:68. 10.1186/1478-811X-11-68 24047437 PMC3851317

[33] Al-Modawi RN, Brinchmann JE, Karlsen TA. Multi-pathway protective effects of MicroRNAs on human chondrocytes in an in vitro model of osteoarthritis. Mol Ther Nucleic Acids. 2019; 17:776–90. 10.1016/j.omtn.2019.07.011 31446120 PMC6716067

[34] Nugent M. MicroRNAs: exploring new horizons in osteoarthritis. Osteoarthritis Cartilage. 2016; 24:573–80. 10.1016/j.joca.2015.10.018 26576510

[35] Dehghani S, Alipoor E, Salimzadeh A, Yaseri M, Hosseini M, Feinle-Bisset C, Hosseinzadeh-Attar MJ. The effect of a garlic supplement on the pro-inflammatory adipocytokines, resistin and tumor necrosis factor-alpha, and on pain severity, in overweight or obese women with knee osteoarthritis. Phytomedicine. 2018; 48:70–75. 10.1016/j.phymed.2018.04.060 30195882

[36] Petersson IF, Boegård T, Saxne T, Silman AJ, Svensson B. Radiographic osteoarthritis of the knee classified by the ahlbäck and kellgren & lawrence systems for the tibiofemoral joint in people aged 35-54 years with chronic knee pain. Ann Rheum Dis. 1997; 56:493–96. 10.1136/ard.56.8.493 9306873 PMC1752423

[37] Kuo SJ, Liu SC, Huang YL, Tsai CH, Fong YC, Hsu HC, Tang CH. TGF-β1 enhances FOXO3 expression in human synovial fibroblasts by inhibiting miR-92a through AMPK and p38 pathways. Aging (Albany NY). 2019; 11:4075–89. 10.18632/aging.102038 31232696 PMC6628998

[38] Wu TJ, Lin CY, Tsai CH, Huang YL, Tang CH. Glucose suppresses IL-1β-induced MMP-1 expression through the FAK, MEK, ERK, and AP-1 signaling pathways. Environ Toxicol. 2018; 33:1061–68. 10.1002/tox.22618 30098273

[39] Wang M, Chao CC, Chen PC, Liu PI, Yang YC, Su CM, Huang WC, Tang CH. Thrombospondin enhances RANKL-dependent osteoclastogenesis and facilitates lung cancer bone metastasis. Biochem Pharmacol. 2019; 166:23–32. 10.1016/j.bcp.2019.05.005 31075265

[40] Liu JF, Lee CW, Tsai MH, Tang CH, Chen PC, Lin LW, Lin CY, Lu CH, Lin YF, Yang SH, Chao CC. Thrombospondin 2 promotes tumor metastasis by inducing matrix metalloproteinase-13 production in lung cancer cells. Biochem Pharmacol. 2018; 155:537–46. 10.1016/j.bcp.2018.07.024 30031810

[41] Lee HP, Chen PC, Wang SW, Fong YC, Tsai CH, Tsai FJ, Chung JG, Huang CY, Yang JS, Hsu YM, Li TM, Tang CH. Plumbagin suppresses endothelial progenitor cell-related angiogenesis in vitro and in vivo. Journal of Functional Foods. 2019; 52:537–44. 10.1016/j.jff.2018.11.040

[42] Lee HP, Wang SW, Wu YC, Lin LW, Tsai FJ, Yang JS, Li TM, Tang CH. Soya-cerebroside inhibits VEGF-facilitated angiogenesis in endothelial progenitor cells. Food and Agricultural Immunology. 2020; 31:193–204. 10.1080/09540105.2020.1713055

[43] Lee HP, Wang SW, Wu YC, Tsai CH, Tsai FJ, Chung JG, Huang CY, Yang JS, Hsu YM, Yin MC, Li TM, Tang CH. Glucocerebroside reduces endothelial progenitor cell-induced angiogenesis. Food and Agricultural Immunology. 2019; 30:1033–45. 10.1080/09540105.2019.1660623

[44] Su CM, Tang CH, Chi MJ, Lin CY, Fong YC, Liu YC, Chen WC, Wang SW. Resistin facilitates VEGF-C-associated lymphangiogenesis by inhibiting miR-186 in human chondrosarcoma cells. Biochem Pharmacol. 2018; 154:234–42. 10.1016/j.bcp.2018.05.001 29730230

[45] Wu KM, Hsu YM, Ying MC, Tsai FJ, Tsai CH, Chung JG, Yang JS, Tang CH, Cheng LY, Su PH, Viswanadha VP, Kuo WW, Huang CY. High-density lipoprotein ameliorates palmitic acid-induced lipotoxicity and oxidative dysfunction in H9c2 cardiomyoblast cells via ROS suppression. Nutr Metab (Lond). 2019; 16:36. 10.1186/s12986-019-0356-5 31149020 PMC6537189

[46] Liu SC, Tsai CH, Wu TY, Tsai CH, Tsai FJ, Chung JG, Huang CY, Yang JS, Hsu YM, Yin MC, Wu YC, Tang CH. Soya-cerebroside reduces IL-1 beta-induced MMP-1 production in chondrocytes and inhibits cartilage degradation: implications for the treatment of osteoarthritis. Food and Agricultural Immunology. 2019; 30:620–32. 10.1080/09540105.2019.1611745

[47] Yang YC, Chiou PC, Chen PC, Liu PY, Huang WC, Chao CC, Tang CH. Melatonin reduces lung cancer stemness through inhibiting of PLC, ERK, p38, β-catenin, and twist pathways. Environ Toxicol. 2019; 34:203–09. 10.1002/tox.22674 30421542

